# Unveiling the Link: The Potential Roles of Vitamin D in Keloid Pathophysiology

**DOI:** 10.1111/exd.70043

**Published:** 2025-02-03

**Authors:** Jaco Kotze, Evangeline Nortje, Alisa Phulukdaree, Mark William Fear, Fiona Wood, Janette Bester

**Affiliations:** ^1^ Department of Physiology, School of Medicine, Faculty of Health Sciences University of Pretoria Pretoria South Africa; ^2^ Burn Injury Research Unit School of Biomedical Sciences Nedlands Western Australia Australia; ^3^ Fiona Wood Foundation Murdoch Western Australia Australia; ^4^ Burns Service WA, WA Department of Health Fiona Stanley Hospital Murdoch Western Australia Australia

**Keywords:** fibroblast, immunomodulatory, keloid, keratinocytes, vitamin D

## Abstract

Keloid disease, a fibroproliferative skin disorder, is characterised by scar tissue growth that extends beyond the original wound boundaries. Despite advancements, current treatments, particularly surgical excision, often result in high recurrence rates, ranging from 45% to 100%. Recent investigations into the role of vitamin D (vit D) in keloids present a promising avenue for novel therapeutic strategies. Studies have highlighted the multifaceted involvement of vit D, including its immunomodulatory effects and influence on key processes such as fibroblast activity, collagen production and extracellular matrix dynamics. Additionally, emerging research has explored the potential impact of vit D on epithelial‐to‐mesenchymal transition and endothelial dysfunction, both of which are implicated in keloid formation and progression. This review consolidates the current evidence linking vitamin D deficiency to keloid pathogenesis, shedding light on potential mechanisms and therapeutic targets. By elucidating the intricate interplay between vit D signalling and keloid development, this study paves the way for innovative treatment approaches that may enhance patient outcomes and mitigate the burden of this challenging dermatological condition.

## 1 Introduction

Keloid disease is a fibroproliferative skin disorder characterised by persistent scar growth beyond the confines of the original wound [1]. This pathological progression is attributed to the continued production of excessive disorganised collagen by hyperproliferative fibroblasts. Keloid genesis is commonly associated with traumatic wounds and inflammatory processes. Keloids usually appear 3 months post‐injury; however, instances of keloid emergence several years after the initial trauma have been documented [2]. A definitive diagnosis of keloid is established when a scar exhibits continuous growth following the initial injury, with no discernible signs of regression. This diagnostic criterion underscores the dynamic nature of keloids and their propensity to extend beyond the expected wound healing trajectory.

Keloids and hypertrophic scars can be clinically and histologically distinguished, although they share some similarities [3]. Keloids manifest in different sizes, characterised by firmness, hyperpigmentation and shiny surfaces [2]. Areas of inflammation and ulceration were evident in the thin epithelium. Predominantly, these manifestations occur in the earlobes, anterior chest, shoulders, upper arms and cheeks [4]. The distribution pattern remains uncertain, with some authors postulating a correlation between skin tension and areas subjected to repeated trauma [5]. In addition to the aesthetic impact of keloids, these conditions cause pruritus and pain [6]. Histologically, keloids exhibit distinctions from hypertrophic scars in the presence of disorganised collagen bundles, absence of nodules and sparse myofibroblasts [7].

Keloids often occur between the first and third decade of life, with a male‐to‐female ratio of 1:2 [8, 9]. It usually presents with a familial pattern and is characterised by an autosomal dominant inheritance with variable expressivity and incomplete penetrance [10]. Keloid disease exhibits heterogeneous prevalence across geographical regions, and the predisposition for keloid formation among individuals with darker skin tones has been collectively confirmed [11, 12, 13, 14, 15]. The incidence ratio in Caucasians is 15:1 which is much lower than the incidence ratio of 5:1 in Asians and dark‐skinned individuals [16]. However, data on incidence is inconsistent across reports and can be challenging to interpret and therefore further work to better understand incidence/ethnicity links will be required [17, 18]. While there are frequent clinical observations of keloids in African populations, specific prevalence data for countries such as South Africa remain unavailable. The proven predilection for darker skin tone groups and associated genetic predispositions, along with other contributing factors discussed in the subsequent sections of this review, emphasise the intricate nature of keloid formation [18].

Although considerable efforts have been made to understand keloid pathophysiology, the underlying cause and optimal treatment have not yet been established [19]. The primary cellular contributors to keloid pathophysiology remain a subject of ongoing debate. Current understanding posits that keloid development is a complex multifactorial process. Notably, various cells are known to be involved in the development of keloids. However, there is a specific focus on inflammatory cells such as lymphocytes, macrophages and mast cells, which appear to play a significant role in the development of keloids. These cells are responsible for the production of key cytokines, including interleukin 6, −17 and transforming growth factor‐beta (TGF β1 and ‐β2), particularly in individuals predisposed to keloid development [5]. These cytokines have been shown to trigger fibroblasts to produce collagen, perpetuating a destructive cycle of collagen synthesis and deposition. This ongoing cycle causes over‐sensitised fibroblasts to recruit neighbouring fibroblasts through the paracrine action of cytokines and through paratensile signalling, which further contributes to keloid formation [20].

Current keloid treatment remains unsatisfactory, as evidenced by recurrence rates ranging from 45% to 100% when surgical excision is used as a standalone modality [2]. The precise factors that contribute to keloid recurrence remain unclear. Numerous treatment modalities have been proposed and are currently in use, including intralesional steroids, 5‐fluorouracil, cryotherapy and bleomycin injections [3]. Surgical excision, often coupled with intralesional steroids and superficial radiotherapy for larger keloids, has demonstrated limited efficacy and provides no definitive solution [2, 21]. Efforts to identify specific markers that can effectively predict patients at risk of keloid formation, along with the development of prophylactic treatment approaches to prevent keloid development, constitute a crucial and imperative area of ongoing research. Despite the ongoing challenges in keloid treatment and the quest for definitive solutions, recent studies exploring the potential involvement of vitamin D (vit D) in keloid formation have opened new avenues for understanding this complex pathology and may pave the way for innovative treatment strategies.

## 2 Exploring the Role of Vitamin D Deficiency and Skin Pigmentation in Keloid Pathophysiology

Recent studies have explored the potential involvement of vit D and its receptor (VDR) in the intricate process of keloid formation [19, 22, 23, 24]. This exploration arises from the observed association between keloid disease and individuals with darker skin tones, as well as the plausible link between vit D deficiency and diminished vit D production in patients with darker skin tones [25]. Vit D deficiency is commonly defined as a serum 25‐hydroxyvitamin D [25(OH)D] concentration below 20 ng/mL (50 nmol/L), with insufficiency classified as levels between 21 and 29 ng/mL (52.5–72.5 nmol/L). A deficiency occurs when there is inadequate dietary intake, insufficient exposure to sunlight, or issues with absorption or metabolism [26]. A meta‐analysis published in 2020 highlighted the high prevalence of vit D deficiency in Africa, particularly among the dark‐skinned population [25]. Additionally, a recent study conducted in the United Kingdom revealed a statistically significant association between keloid disease, hypertension and dark skin [27].

Vitamin D is predominantly synthesised by exposure to sunlight [28]. Consequently, skin pigmentation, the main regulator of ultraviolet (UV) B penetration, is inversely associated with vit D3 production in the skin [29]. Individuals classified as Fitzpatrick type VI, with a protection factor of 15% and 99% absorption of UVB radiation, experience a 99% decrease in vitamin D3 production [30, 31].

## 3 Known Functions of Vitamin D and Its Potential Role in Keloids

Historically recognised as a key regulator of calcium and phosphorous absorption and metabolism [32], vit D has transcended its primary role in skeletal health. The presence of VDR signalling in various cells throughout the body suggests its involvement in multiple physiological pathways. Vitamin D has been linked to modulation of cell growth, proliferation, immune function, regulation of hormone secretion and gene expression [33]. Extending beyond these fundamental roles, vit D has been associated with the prevention and treatment of a diverse range of health conditions. These include cardiovascular diseases, autoimmune disorders, oncological conditions and infectious diseases [33], highlighting the far‐reaching impact of vit D on human health. Vitamin D is known to exert anti‐fibrotic, anti‐inflammatory and anti‐proliferative effects through its regulation of VDR within the vit D signalling pathway [23].

### 3.1 Vitamin D Receptor and Vitamin D Levels

Currently, there is a paucity of clinical research on the correlation between keloids and vit D levels. Nonetheless, previous studies have confirmed that individuals with severe burns frequently develop keloids, and a considerable proportion of these patients suffer from vit D deficiency owing to a range of factors [34, 35, 36].

A large study by Yu et al. showed that keloid patients have lower serum vit D levels than controls, and they discovered that a serum vit D threshold of 16.1 ng/mL effectively differentiated between individuals with keloids and the control group [37]. Furthermore, a recent study by El Hadidi et al. also showed lower serum vit D levels in keloid patients; however, no association was found between Fitzpatrick skin type and serum vit D levels [38, 39]. This study also found lower VDR expression in keloid patients in both keloids and unaffected skin of keloid patients, but no association between VDR expression and serum vit D levels [39]. In contrast to the aforementioned study, reduced expression of VDR in the epidermis of keloids, along with diminished rates of nuclear localization, was observed in individuals with darker skin tones, specifically African American patients, in comparison to those with lighter skin tones, such as Caucasian patients [22]. Similarly, Gong et al. showed that VDR expression was systemically decreased, and although there was no correlation with systemic vit D levels, the main finding was an increased risk of keloid disease [40]. Damanik et al. demonstrated a correlation between keloid severity and lower serum 25‐hydroxyvit D (25(OH)D) levels. Furthermore, their findings indicated reduced VDR expression in keloid epidermis in comparison to normal skin in keloid patients, which suggests the potential role of vit D deficiency and reduced VDRs in keloid formation [22].

**FIGURE 1:**
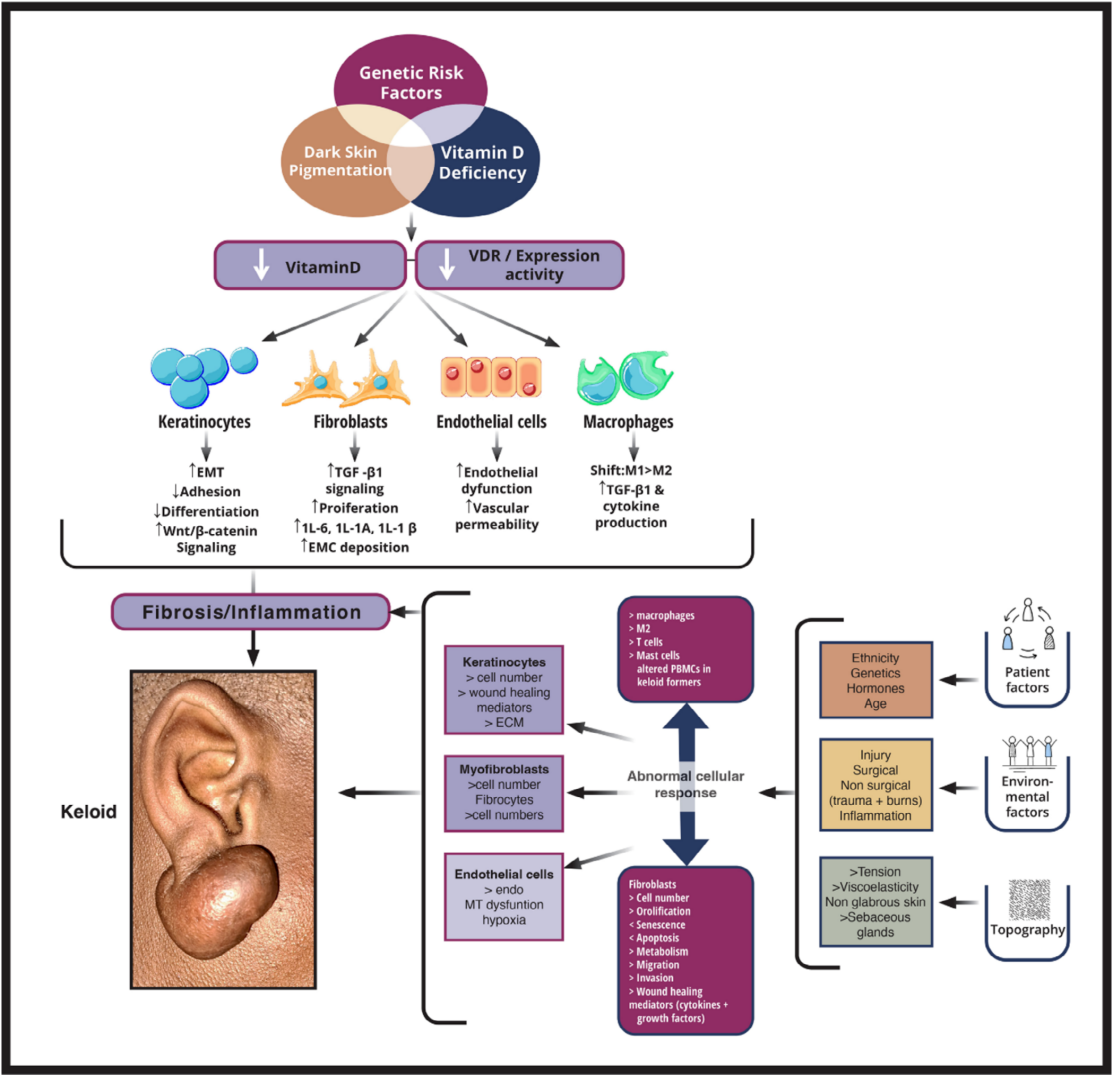
Generally, the proposed pathophysiology of keloid formation involves multiple factors, including patient‐related, environmental, and topographical aspects. These factors collectively impact various cell types, including keratinocytes, endothelial cells, macrophages, and fibroblasts, contributing to the development of keloids. The depicted hypothesis highlights the potential role of vit D signalling through the vit D receptor (VDR) in this process. The key contributors to the development of keloid disease include genetic risk factors, dark skin pigmentation, and vit D deficiency. The reduction in circulating vit D and VDR levels influences the mentioned cell types, inducing a pro‐inflammatory and pro‐fibrotic state. This cascade ultimately initiates and progresses keloid formation. Diagram created by Creative Studio adapted from Hahn et al. [43] ECM, Extracellular matrix; EMT, Epithelial‐to‐mesenchymal transition; M, Macrophage.

In general, the majority of studies indicate that there is diversity in the expression of VDR, and individuals with keloids often exhibit lower systemic vit D levels. Nevertheless, deriving statistically robust conclusions remains challenging, primarily because of the limited number of studies aiming to establish a correlation between vit D and keloid formation. Additionally, the challenges extend to constrained sample sizes, insufficient follow‐up and scarcity of comprehensive clinical studies [37, 38, 39, 41, 42]. Assessing optimal vit D levels also proves challenging because of the complexity of the vit D pathway, leading to the formation of several active forms [43, 44, 45]. Current methods for measuring serum vit D levels have limitations, and it remains debatable whether various reported disease processes are linked to vit D deficiency [46]. Furthermore, the ideal dosage and supplementation method for vit D, as well as the optimal level for deriving health benefits, remain subjects of ongoing debate. The multifaceted interplay between vit D and keloid formation, along with other potential mechanisms, is summarised in Figure 1 [47]. The interaction between vit D and its receptor is central to its physiological effects, and this relationship is modulated by the availability of its active ligand, 1,25‐dihydroxyvitamin D3, as well as the enzymes responsible for its synthesis and degradation.

### 3.2 Ligand

In the context of vit D, the ligand in the vit D signalling pathway typically refers to its active form, 1,25‐dihydroxyvitamin D3 (calcitriol), which binds to VDR [22].

As a transcription factor, ligand‐bound VDR, a member of the steroidal nuclear receptor superfamily, modulates cellular processes in various cell types [22, 48, 49]. Initiated by the binding of vit D3 to VDR, the resulting vit D3/VDR complex that interacts with vit D response elements (VDREs) in target gene promoter regions, thereby regulating their transcription [50]. This complex, favouring binding with the retinoid X receptor (RXR), triggers an epigenetic effect, altering the transcription of immune and inflammatory genes without modifying DNA [50]. However, literature shows that the nuclear localization of VDR is markedly diminished in the keloid epidermis compared to that in the normal skin. This phenomenon may contribute to attenuated expression of downstream target genes [21]. This reduced nuclear localization may play a role in keloid pathology by amplifying the response of mothers against decapentaplegic (Smad) TGF‐β1 signalling, influencing inflammation and regulating epithelial‐to‐mesenchymal transition [22, 48, 49]. Remarkably, the nuclear localization of VDR increases upon vit D treatment, suggesting its potential application in the treatment of abnormal scarring and the prevention of keloid formation [51]. While the VDR is essential for the transcriptional effects of vit D, its activation depends on the enzymatic conversion of vit D precursors into their bioactive forms.

### 3.3 Enzymes

**FIGURE 2:**
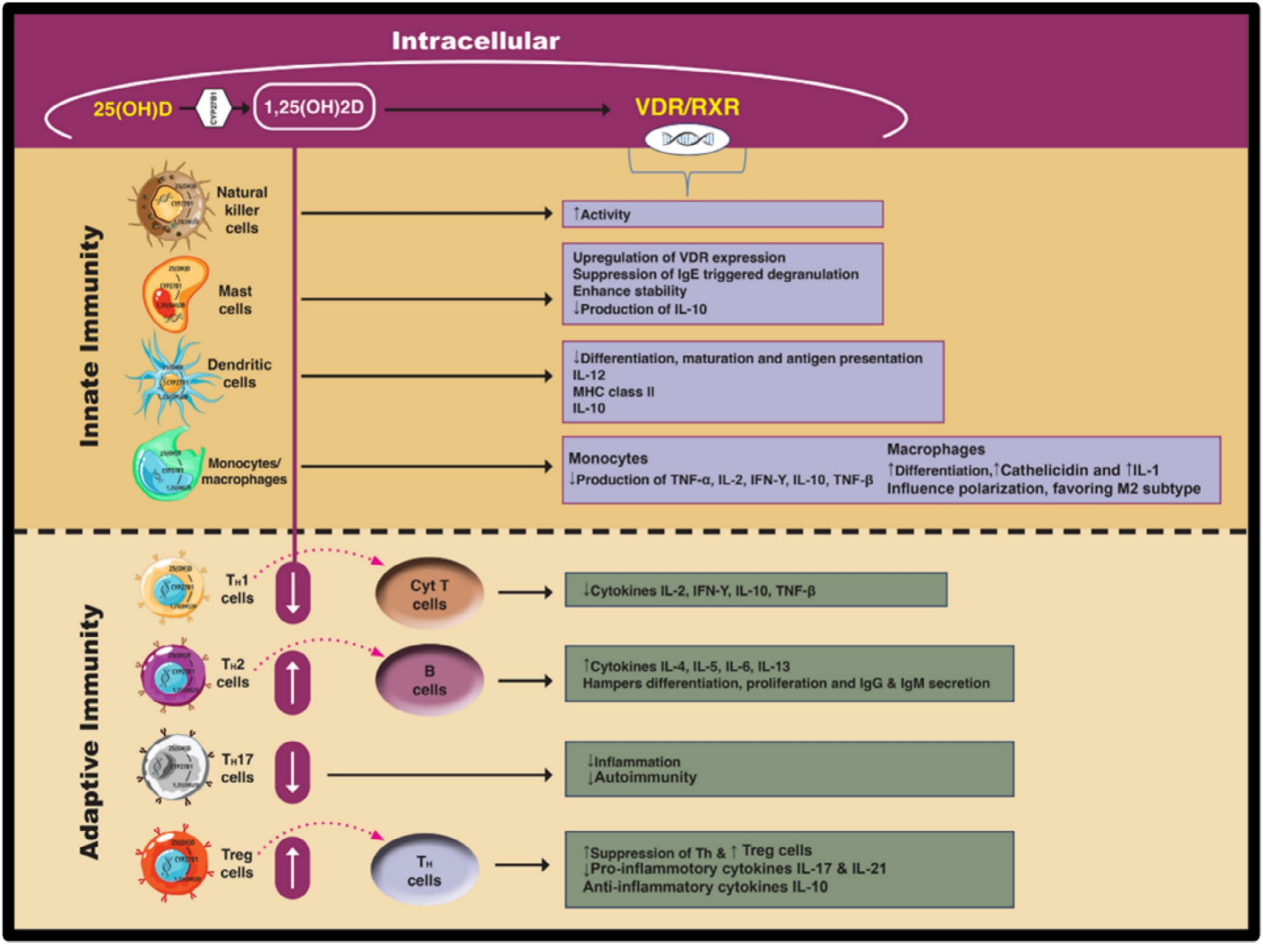
Vitamin D has immunomodulating effects on the adaptive and innate immune systems by altering the behaviour, number as well as cytokine/ growth factor expression of various immune cells. Through its signalling pathway, seen in all these cells, initiated by the binding of vit D3 (1,25(OH)2D) to VDR, the resulting vit 1,25(OH)2D /VDR complex interacts with vit D response elements (VDREs) in target gene promoter regions, thereby regulating their transcription. This complex, favouring binding with retinoid X receptor (RXR), triggers an epigenetic effect, altering the transcription of immune and inflammatory genes. Purple dotted arrows indicate activation of the specific immune cells, and the solid black lines indicate the epigenetic effects of the vit 1,25(OH)2D /VDR complex binding to RXR. Created by Creative Studio and adapted from Cho et al. [32]. MHC, Major histocompatibility complex.

By studying the immunomodulatory functions of vit D, researchers have gained a deeper understanding of the crucial role of extrarenal 1α‐hydroxylase (CYP27B1) in vit D physiology [52]. This enzyme is expressed in diverse immune cell types including macrophages, dendritic cells, lymphocytes and monocytes (Figure 2) [52, 53]. Unlike its renal counterpart, it functions primarily under the regulation of immune signals rather than via calcium homeostatic mechanisms.

CYP27B1 is also expressed in skin cells, including fibroblasts, keratinocytes and endothelial cells, where it facilitates the local conversion of 25‐hydroxyvitamin D to its active form, vit D3. The impact of vit D3 is brief because it stimulates the enzyme 24‐hydroxylase (CYP24), which swiftly breaks down the hormone. This process triggers a sequence of oxidised side‐chain byproducts, eventually leading to a decrease in activity [54]. Reduced or abnormal expression of CYP27B1 in keloid tissue could lower vit D3 levels, whereas lower levels of CYP24 or selective inhibitors of CYP24 could slow down the metabolism of vit D3, potentially disrupting the pathways that help drive keloid formation. Genetic variations in the CYP27B1 and CYP24 genes may also promote individual susceptibility to keloids, as differences in enzyme activity can alter vit D3 levels and thereby impact the regulation of fibroblast and immune cell activity.

However, the current research is insufficient to confirm these findings in the context of keloid pathophysiology, leaving these mechanisms largely theoretical.

### 3.4 Known Immunomodulatory Effects of Vitamin D

Vitamin D exerts diverse effects on immune cells through its signalling pathway. It hampers B‐cell differentiation, proliferation and immunoglobulin secretion [55]. Moreover, vit D induces a shift from a T Helper cell 1 (TH1) to a T Helper cell 2 (TH2) phenotype by suppressing T cell proliferation and inhibiting T cell maturation, steering away from the T17 phenotype. It is evident from the literature that the TH2 inflammatory axis plays an integral role in keloid pathogenesis, with IL‐4 and IL‐13 serving as key mediators [56, 57]. This axis is characterised by the overexpression of fibrotic cytokines, which contribute to excessive collagen production observed in keloids. Interestingly, recent epidemiological data provided by Ung et al. further support the association between TH2‐skewed inflammation and keloid formation [26].

Additionally, it plays a role in increasing the number of T regulatory (TREG) cells, the primary T cells that inhibit immune reactivity [52]. These alterations, facilitated by vit D, reduce the production of pro‐inflammatory cytokines, such as interleukin (IL)‐17 and IL‐21, while boosting anti‐inflammatory cytokines, such as IL‐10. Furthermore, vit D impedes the production of cytokines (TNF‐α, IL‐12, IL‐8, IL‐6 and IL‐1) by monocytes. Finally, vit D inhibited the differentiation and maturation of dendritic cells [55].

This suggests that vit D may play a supportive role in modulating both innate and adaptive immune responses to tissue injury via numerous signalling pathways, as depicted in Figure 2 [53, 58, 59, 60].

### 3.5 Vitamin D's Immunomodulatory Effect on Key Immune Cells in Keloid Disease

#### 3.5.1 T Lymphocytes

To date, abundant evidence has substantiated the involvement of the innate and adaptive immune systems in the pathophysiology of keloid disease. With this in mind, the role of T lymphocytes or T cells in keloid formation is multifaceted and diverse, and is inhibited or promoted by the various subsets of T cells involved. T cells can modulate wound healing and contribute to the promotion of keloid formation through the release of different cytokines [5]. T Helper (TH) 1 cells are characterised by the expression of IL‐2, interferon (IFN)‐γ, IL‐10 and TNF‐β. In contrast, TH2 clones are known for express IL‐4, IL‐5, IL‐6 and IL‐13 [61]. The TH1 response in keloids decreased with lower production of the anti‐fibrotic cytokine IFN‐γ. Interleukin 4 and IL‐13 (TH2 response) are overexpressed in patients with keloids [5]. These cytokines are potent fibrotic markers [5]. It is important to note that the function of T cells is intricate and not definitively understood, as their behaviour can vary in altered microenvironments.

In the early stages of wound healing, pro‐inflammatory cytokines predominate, whereas in the late stages, there is a shift towards an abundance of anti‐inflammatory cytokines. When there is an increased and lengthy pro‐inflammatory milieu, aberrant scarring and possible keloid formation might follow [62]. Various pro‐inflammatory cytokines, including IL‐6, IL‐8, IL‐18 and chemokine like factor‐1 (CKLF‐1), are significantly elevated in keloid tissue, and IL‐8 and IL‐17 are elevated in the peripheral blood of keloid patients [5, 63, 64]. A recent study by Nangole et al. also showed elevated levels of various pro‐inflammatory cytokines in the plasma of keloid patients compared to those in the control group [62]. The question remains as to whether these are the consequences or causes of keloid formation.

It has been shown that CD4^+^ and CD3^+^ T cell counts are higher in keloid lesions than in normal skin [65]. Vitamin D receptors expressed on CD4^+^ cells have a high affinity for vit D and are known to influence the immune system by targeting CD4^+^ cells [66]. It is theorised that the VDR transcripts found in TH17 cells contribute to the IL‐6/IL‐17 axis, which may promote primitive cells in keloid lesions [67]. Therefore, sustained amounts of pro‐inflammatory cytokines may influence keloid formation. Supporting this, keloids have been hypothesized to arise from chronic inflammation of the reticular dermis [68]. Emerging evidence suggests that vit D possesses robust immunomodulatory effects, thereby holding the potential to promote keloid formation.

As mentioned, the anti‐inflammatory effects of vit D alter the cytokine profile towards a TH2 phenotype, regulate the expression of chemokines and increase the secretion of IL‐10, reducing TH1 cytokines, thus curbing nuclear factor kappa‐light‐chain of activated B cells (NF‐κB) activity and potentiating the actions of glucocorticoids [52]. The NF‐κB signalling pathway is inhibited by 1,25(OH)_2_D through VDRs [69]. Lim et al. showed that the signal transducer and activator of transcription (STAT)‐3 pathway is activated in keloid tissues [70]. This pathway modulates inflammation and fibrosis and regulates cell apoptosis, proliferation, migration and differentiation through various cytokines, including IL‐6, IL‐7, IL‐9, IL‐10, IL‐15, IL‐23, IL‐21 and IL‐11 [71]. Activation of the vit D pathway could be a mechanism to decrease the activation of the STAT‐3 pathway [72].

#### 3.5.2 Macrophages

The role in keloid pathologies is intricate and multifaceted. Historically recognised for their phagocytic role in debris clearance and tissue remodelling [5] macrophages are divided into M1 and M2 types, both critical in wound healing [73]. Macrophages function as antigen‐presenting cells by releasing various cytokines and growth factors that enhance tissue repair, ultimately stimulating an adaptive immune response [73, 74]. M1 macrophages are associated with the inflammatory phase of wound healing, generally mitigating pro‐inflammatory responses. M2 macrophages contribute to decreased inflammation and improved epidermal regeneration [75]. M1 macrophages predominantly release pro‐inflammatory cytokines, such as IL‐12 and TNF‐α, while M2 macrophages primarily release anti‐inflammatory cytokines, such as IL‐2 and TGF‐b3. Bagabir et al. demonstrated an increase in macrophages in keloidal tissue, and Shaker et al. hypothesized paracrine activity between macrophages and fibroblasts in keloid tissue through cytokines [76, 77]. Another study by Jin Q and colleagues revealed higher activation levels of macrophages in keloid tissue than in the normal skin [65]. These macrophages were predominantly polarised towards M2 subtypes and exhibited increased transcription and protein expression of TGF‐β, IL‐12, IL‐10 and inducible nitric oxide synthase (INOS) [65]. However, the number of macrophages in these keloid specimens varied in the same person and was not elevated in all [65].

Vitamin D influences the polarisation of macrophages, favouring the M2 subtype and enhancing the synthesis of anti‐inflammatory cytokines, potentially suppressing excessive pro‐inflammatory responses observed in keloid disease [78]. Notably, this is particularly intriguing in the context of keloid disease, where M2 macrophages are the predominant phenotype in established cases [5].

One may speculate that during the initial stages of wound healing in patients at risk of developing keloids, M1 macrophages may predominate. A critical question that arises is whether the balance between M1 and M2 macrophages becomes completely dysregulated in the initial stages of wound healing, resulting in excessive activation of fibroblasts and other immune cells [79]. This imbalance results in elevated collagen production, thereby playing a role in the formation of keloids.

If this hypothesis holds true, the introduction of vit D could potentially restore a more physiological balance between M1 and M2 macrophages in the early stages of keloid formation. Consequently, it can prevent an excessive inflammatory response, ultimately mitigating the excessive fibrosis characteristic of keloid formation.

#### 3.5.3 Mast Cells

Mast cells, vital players in the innate immune response, stimulate fibroblast proliferation and collagen synthesis, contributing to wound healing and possibly keloid formation [80].

Additionally, mast cells produce cytokines that can further promote fibroblast activity. Histamine and heparin secreted by mast cells contribute to pruritus and increased vascularity in keloids, respectively. A link between allergic symptoms and hypertrophic scarring and keloids has also been reported [5].

Vitamin D exerts multiple effects on mast cells, including upregulation of VDRs on mast cells, suppression of IgE‐triggered mast cell degranulation, enhanced mast cell stability and increased production of IL‐10 [81]. The close relationship between mast cells and vit D helps explain the contradictory actions attributed to mast cells. Although mast cells are traditionally believed to primarily trigger inflammation and allergic reactions, recent discoveries indicate that they can mitigate cellular damage and inflammation caused by UVB irradiation. Under specific circumstances, mast cells produce cytokines such as IL‐10, which have the potential to alleviate skin damage resulting from prolonged exposure to UVB radiation [81].

Additionally, mast cells can convert 25OHD3 to the active form of vit D (1α,25(OH)2D3) through CYP27B1 [81]. Vitamin D3 derivatives generated by mast cells reduce the production of pro‐inflammatory mediators by binding to the VDR. The application of vit D3 metabolites to the skin surface leads to a notable reduction in skin inflammation, involving interactions between mast cells and VDRs, as well as mast cells and CYP27B1. Therefore, vit D exerts anti‐inflammatory effects through mast cell function. These cells also have the ability to metabolise 25OHD3, leading to the inhibition of IgE‐mediated mast cell activation in both in vivo and in vitro experiments [81].

The intricate interplay among immune cells, cytokines and the microenvironment forms the foundation of keloid pathogenesis. In the preceding sections, we explored how T lymphocytes, macrophages, and mast cells uniquely contribute uniquely to the immune landscape in keloid disease. Vitamin D influences these cells by modulating their phenotype and cytokine profiles, which significantly affects the inflammatory milieu.

### 3.6 Other Known Effects of Vitamin D in Keloids

#### 3.6.1 Fibroblast‐Mediated Keloid Formation and the Link to Vitamin D

Fibroblasts play a central role in tissue repair and wound healing by producing extracellular matrix (ECM) components such as collagen [82]. In keloid disease, fibroblasts demonstrate significant proliferative potential, elevated migration and invasion abilities, and increased extracellular matrix build‐up, all of which play a role in keloid formation. Historically, fibroblasts have been considered a homogeneous population of spindle‐shaped cells. However, recent evidence reveals that fibroblasts are a diverse population with variations in both structure and function [83]. Single‐cell RNA sequencing (scRNA‐seq) has provided a valuable tool for investigating the heterogeneity of skin fibroblasts under both normal and pathological conditions. scRNA‐seq has indicated that fibroblasts in the healthy human dermis can be classified into several distinct subgroups [83]. Deng et al. demonstrated that by employing scRNA‐seq along with further clustering analysis, fibroblast subtypes can be classified into four distinct groups: secretory‐papillary, secretory‐reticular, mesenchymal, and proinflammatory. The mesenchymal subtype was identified as the most dominant in keloid tissue and, interestingly, exhibited gene expression linked to skeletal development, bone formation, and osteoblast maturation. Another interesting discovery was that mesenchymal fibroblasts play a crucial role in the overexpression of collagen in keloids via *POSTN* periostin (POSTN), a protein encoded by the *POSTN* gene [83]. This protein plays a key role in tissue remodelling and fibrosis. Interestingly, an in vitro study showed that maxacalcitol, a vit D analog, reduces periostin expression in cultured mouse fibroblasts stimulated by TH2 cytokines and TGF‐β [84]. Further research is required to clarify the connection between vit D and mesenchymal fibroblast subtypes.

#### 3.6.2 The Role of Vitamin D on the Direct Actions of Fibroblasts on Keloids

The involvement of vit D in keloid formation has been recognised, with evidence pointing to its contribution through various mechanisms [32]. One such mechanism involves modification of gene expression, which in turn affects collagen and ECM synthesis in fibroblasts [85]. Vitamin D may play a crucial role in keloid regression, as evidenced by a dose‐dependent reduction in fibroblast proliferation and a significant drop in collagen 1 expression [79]. Additionally, Dobak et al. demonstrated that vit D3 hinders the proliferation of dermal fibroblasts. However, in contrast to the above‐mentioned study, vit D3 in their study increased the production of collagen types 1 and 3 independently of fibroblast proliferation in vitro [86].

Vitamin D also interacts with various signalling pathways involved in fibroblast activation by regulating the expression of receptors and downstream signalling molecules within fibroblasts. One such pathway is the TGF‐β pathway, in which TGF‐β acts as a key cytokine that promotes fibrosis by activating the fibroblasts. VDR has been shown to inhibit fibroblast activation via this pathway [41].

Transcription factors of the NF‐κB family modulate many key inflammatory genes and have been shown to be activated in keloid fibroblasts [69]. In another in vitro study, the introduction of vit D3 to incubated keloid fibroblasts reduced the expression of collagen type 1, fibronectin, and α‐SMA [87]. This effect was achieved through inhibition of the vit D‐mediated TGF‐β pathway [87].

#### 3.6.3 Indirect Fibroblast‐Mediated Keloid Formation and the Link to Vitamin D

Keloid formation likely involves a complex interplay between various cellular and molecular factors. Fibroblasts, in response to signals from inflammatory cells, such as macrophages and lymphocytes, can become activated and produce excessive amounts of collagen and other ECM components. Transforming growth factor β1, among other cytokines previously mentioned, is known to stimulate fibroblasts and promote collagen synthesis, contributing to the characteristic overgrowth of scar tissue observed in keloids [71, 88].

This association with immune modulation suggests that keloid disease is intricately linked to the immune processes. Therefore, by changing the microenvironment, vit D may indirectly inhibit fibroblast activity and mitigate fibrotic processes. An in vitro study demonstrated the anti‐inflammatory effects of TREGs in inhibiting collagen expression in fibroblasts [5]. In contrast, TREGs isolated from fibroblasts increased the expression of collagen in fibroblasts [71]. In addition, studies showed that TREGs can stimulate fibroblasts through the secretion of IL‐10 and TGF‐β [89, 90]. The increased presence of TREGs in keloids raises inquiries regarding the causative factors behind their increased numbers.

#### 3.6.4 The Role of Vitamin D in Extracellular Matrix Regulation During Keloid Formation

As mentioned, the inflammatory milieu observed during keloid formation stimulates fibroblasts, leading to excessive ECM production [5, 55]. Evidence supports the hypothesis that fibroblast activity and ECM production increase when exposed to a sustained inflammatory environment [91].

Previous studies have provided compelling evidence regarding the impact of vit D and its analogues on fibroblast proliferation and collagen type 1 expression [92, 93]. Moreover, vit D has been shown to augment the expression of caspase‐3, a pro‐apoptotic factor, while simultaneously diminishing the expression of BCL‐2, an anti‐apoptotic factor. Caspases play a pivotal role in programmed cell death, whereas BCL‐2 serves as a safeguard against cell death. This discovery holds particular relevance given the observation that keloid fibroblasts, in comparison with their normal skin counterparts, exhibit resistance to apoptosis, potentially contributing to the development of keloid pathology [94]. Additionally, VDR exerts a negative regulatory effect on the TGFβ/Smad signalling pathway, effectively impeding collagen production. Notably, the expression of VDR is lower in the peripheral blood lymphocytes of individuals with keloid disease as well as in the epidermis of keloid lesions [24, 40]. A study by Lee et al. showed that vit D3 inhibits ECM production and the differentiation of myofibroblasts was attenuated through a TGFβ/Smad‐dependent signalling pathway. A dose‐dependent reduction in the expression of α‐SMA and fibronectin was observed. Fibronectin is a key component of the ECM, and α‐SMA is a marker of myofibroblasts. Myofibroblasts are the main ECM‐secreting cells in fibrosis and wound healing [95]. While there is widespread acceptance of the pivotal role of myofibroblasts in physiological and pathological scarring, their contribution to keloid formation remains a subject of considerable debate in the literature [96].

#### 3.6.5 Vitamin D, Endothelial Dysfunction, and Keloid Formation

Another potential mechanism is the association of vit D with endothelial dysfunction and keloid formation. It has been proposed that an increase in vascular permeability due to dysfunction in the endothelium could increase a local inflammatory response in the skin which can ultimately lead to hypertrophic scars and keloid formation [97]. Indicators such as an elevated augmentation index and decreased reactive hyperemia index suggest endothelial dysfunction in keloids, while sequencing studies on keloid patients have revealed disrupted angiogenesis‐related pathways [98, 99, 100]. Endothelial dysfunction has also been associated with vit D deficiency, particularly in individuals with darker skin, who also exhibit a higher prevalence of cardiovascular disease [101]. Vitamin D mitigates endothelial dysfunction by suppressing pro‐inflammatory cytokines, reducing nitric oxide (NO) production, and inhibiting oxidative stress in endothelial cells [102].

#### 3.6.6 Role of Vitamin D in the Epithelial‐To‐Mesenchymal Transition and Keloid Keratinocytes

Vitamin D signalling is also potentially linked to keloid formation through its involvement in epithelial‐to‐mesenchymal transition (EMT), a phenomenon associated with keloids and organ fibrosis [103]. Epithelial‐to‐mesenchymal transition is a biological process in which epithelial cells transform into mesenchymal cells, gaining the ability to produce the ECM [104]. This transformation is driven by paracrine signalling, in which cells release signalling molecules, such as growth factors and cytokines, that promote or inhibit nearby cells in the tissue environment [104]. In keloid formation, fibroblasts, keratinocytes, and immune cells secrete various cytokines and growth factors that act in a paracrine manner to stimulate adjacent cells [104]. This paracrine communication creates a positive feedback loop that promotes continuous fibroblast activation and collagen deposition [104].

Keloids are believed to remain in a persistent proliferative phase of wound healing, resembling a partial EMT with an impaired shift towards the remodelling phase [103, 105]. In typical wound healing, EMT‐like characteristics naturally reverse, resulting in reduced cellular proliferation and ultimately facilitating the transition to the remodelling phase. Clinically, this resembles a wound that is closed or epithelized, and the scar is continuously undergoing remodelling [106]. Keloid keratinocytes show abnormal expression of genes that affect the differentiation, migration, and adhesion of these keratinocytes [105].

This gene expression pattern resembles that of EMT, which is commonly observed in the metastasis of cancer cells. However, keloid keratinocytes are thought to have incomplete EMT, which leads to an imbalanced and excessive wound healing response. During EMT, epithelial cells gain features of mesenchymal cells, which include a decrease in adhesion, an increase in migration, and a change in the mesenchymal transcriptional profile [103]. Previous research has indicated that keloid keratinocytes exhibit reduced adhesion in vivo and heightened migration in vitro which resembles EMT‐like characteristics [107]. Keloids may have several features resembling EMT, including the atypical expression of genes involved in Wnt/β‐catenin, TGF‐β1, and Sonic Hedgehog signalling [47].

The vit D signalling pathway has been shown to hinder EMT in various cell types. This is achieved by modifying the target genes associated with epithelial differentiation and cell adhesion as well as inhibiting key triggers of EMT, including Wnt/β‐catenin, TGF‐β1, and Sonic Hedgehog signalling [108, 109].

## 4 Vitamin D, Epithelial Integrity, and Barrier Function

Vitamin D is essential for establishing a strong epithelial barrier in skin. Studies have shown that topical application of vit D3 can improve a disrupted permeability barrier, such as when it is compromised by corticosteroids or sodium lauryl sulphate [110, 111]. This effect occurs because vit D increases the production of structural proteins in the outer layer of the skin. Additionally, vit D regulates the production of long‐chain glycosylceramides, which are critical for the formation of the lipid barrier in the skin [112]. Research also indicates that mice lacking VDR have poor skin barrier function due to decreased transport of glycosylceramides into lamellar bodies, resulting in reduced lipid levels [112]. When epithelial integrity is compromised owing to low vit D levels, the skin becomes more permeable, allowing bacteria and inflammatory mediators to penetrate more easily [112]. This permeability can set the stage for a chronic inflammatory response, activating fibroblasts and other skin cells, which then produce excess collagen and other extracellular matrix components, ultimately driving keloid formation.

## 5 Major Open Questions

Could vit D deficiency be more prevalent among individuals with darker skin, particularly those of African descent, or is it a transethnic issue? In a recent UK cohort, an association between vit D deficiency and diverse ethnic populations could not be established [27].

Could vit D deficiency predispose individuals with dark skin to be more prone to developing keloids?

Will vit D has an immunomodulatory effect on immune cells that can be used to treat keloid disease.

An investigative study should be conducted to specifically address the vit D status among individuals with dark skin who suffer from keloids. This comprehensive study aimed to delve into the intricate functioning of specific immune cells implicated in wound healing, comparing those with and without vit D deficiency. By scrutinising these factors, future studies should endeavour to elucidate the pathophysiological impact of vit D on keloid formation, providing invaluable insights into this complex phenomenon.

## 6 Conclusions and Perspectives

Vitamin D has multiple roles relevant to keloid pathology, influencing processes such as epithelial‐to‐mesenchymal transition (EMT), immune activity, epithelial integrity and fibroblast‐mediated extracellular matrix (ECM) production. However, it remains uncertain which, if any, of the above roles of Vit D are critical in keloid treatment or can be effectively targeted for intervention.

Additionally, it is unknown whether a decrease in VDR expression and nuclear localization are associated with deficient serum vit D levels. Furthermore, it is unknown whether vit D supplementation mitigates keloid formation. The role of vit D as a systemic therapy versus local therapy needs to be studied, as well as how such therapy will be prescribed. Furthermore, given the intricate and multifaceted nature of the mechanisms underlying keloid formation, it is unlikely that vit D alone would suffice as a standalone therapy for keloid management. Treatment strategies should also be tailored to consider pigmentation of the patient's skin and implement preventive measures and appropriate treatment strategies for keloids in patients with skin colour.

Further basic scientific studies and translational research are imperative to ascertain the efficacy of vit D supplementation or targeted therapies as strategies for preventing or treating keloids. An extensive investigation into the molecular mechanisms inhibited or promoted by vit D coupled with translational efforts could pave the way for the development of novel therapeutic interventions. This research trajectory holds promise for advancing our understanding of keloid pathogenesis and informing the development of effective strategies for mitigating or preventing the formation and progression of keloid lesions.

## Author Contributions

J.K., concept development; data collection and manuscript writing. JB, MF, F.W and E.N., data collection; manuscript writing and editing. A.P., manuscript writing and editing.

## Ethics Statement

This review forms part of a larger study that has received ethical clearance from the Research Ethics Committee from the University of Pretoria—456/2022.

## Conflicts of Interest

The authors declare no conflicts of interest.

## Data Availability

The data that support the findings of this study are available on request from the corresponding author. The data are not publicly available due to privacy or ethical restrictions.
